# Acute Portal Vein Thrombosis Secondary to COVID-19 Vaccination

**DOI:** 10.7759/cureus.26825

**Published:** 2022-07-13

**Authors:** Matthew L Repp, Seth Cohen, Caitlin Kibbey

**Affiliations:** 1 Emergency Medicine, University of Arizona, Tucson, USA; 2 Emergency Medicine, University of Texas-Southwestern, Dallas, USA

**Keywords:** vaccine, coronavirus, covid-19, oral contraceptive, portal venous thrombosis

## Abstract

Portal vein thrombosis (PVT) is a partial or complete occlusion of the hepatic portal vein most frequently seen in patients with cirrhotic liver disease. Various non-cirrhotic conditions including inherited prothrombic blood disorders, neoplasms, and inflammatory diseases create hypercoagulable states that predispose individuals to blood clotting. Rarely does an exhaustive workup leave the etiology of a PVT unknown or unclear, and even more uncommon is a potential new etiology suspected. Our patient is a 34-year-old female, with no significant risk factors for pathologic clotting, who was diagnosed with an acute PVT several days after receiving the Moderna coronavirus disease 2019 (COVID-19) vaccine.

## Introduction

According to Virchow’s triad, the three most important factors that bring about pathologic clotting are venous stasis, endothelial injury, and predisposing states to hypercoagulability [1]. Venous thrombi most commonly form in the deep veins of the lower limbs but can also affect other vasculature including the portal venous system. Portal venous thrombosis is a rare vascular disorder most often caused by liver cirrhosis; less frequent etiologies are non-cirrhotic and include neoplasms as well as inherited or acquired hypercoagulable disorders [2]. Nearly 75% of the liver's blood supply is done by the portal vein that drains the gastrointestinal organs via the superior mesenteric, inferior mesenteric, splenic, and gastric veins [3]. An impediment of portal blood flow can lead to parenchymal extinction, which consists of hepatocellular death and pathologic remodeling of liver parenchyma due to tissue hypoxia. In an acute setting such as the emergency department (ED), this most frequently manifests with abdominal pain (91%) and fever (53%) and less frequently with ascites (38%) [4]. Ensuing consequences of PVTs include portal hypertension, hepatic dysfunction, and intestinal infarction.

The novel coronavirus disease 2019 (COVID-19) virus has been known to cause multiple sequelae due to inflammatory processes causing residual organ damage, yet the effects of the COVID-19 vaccine are continually being investigated. Currently, there is equivocal and speculative data suggesting portal venous thrombosis as a side effect of COVID-19 vaccination. As newly introduced vaccines continue to be widely implemented, it is important to identify and inform the medical community and public about potential side effects that may arise in patients with and without comorbidities.

## Case presentation

A 34-year-old female presented to the emergency department (ED) for shortness of breath and abdominal pain. Her past medical history as obtained from the patient was significant for polycystic ovarian syndrome (PCOS) and hypothyroidism. The patient states that she received her second Moderna COVID-19 vaccination five days prior to the ED visit, and she started to have shortness of breath and abdominal discomfort the next morning. Her dyspnea was worsened with exertion and relieved with rest but was otherwise constant. She endorsed associated nausea, diarrhea, subjective fever, lightheadedness with standing, and headache but denied vomiting, cough, sore throat, and urinary symptoms. She denied any sick contacts, recent travel, antibiotic use, recent hospitalizations, surgeries, or prior blood clots. Her social history included casual cigarette usage which she stopped approximately ten years ago and social alcohol consumption. She denied recreational drug use. She reported being sexually active with a male partner and had taken combined oral contraceptive pills (OCPs) consistently for the past 6 weeks. She used OCPs intermittently in the past. She denied being vaccinated for influenza.

Initial vitals were within normal limits (WNL). She had a blood pressure of 138/96, pulse of 71 beats per minute (pm), temperature of 36.7 °C, respiration of 19 breaths per minute, SpO2 of 99%, and BMI of 33.28. Upon examination, the patient’s lungs were clear to auscultation without wheezing, crackles, or rhonchi and she did not appear to be in respiratory distress. She had dry oral mucosa on presentation. Upon abdominal examination, she had tenderness to palpation diffusely across her upper abdomen, worse in the mid-epigastric and right upper quadrant (RUQ). She was negative for rebound tenderness, guarding, and peritoneal signs. Her abdomen was non-distended. No other significant abnormalities were noted. 

Point of care (POC) glucose, POC Hgb, and electrocardiogram (EKG) were obtained at triage and the results were without significant abnormality. Basic labs were ordered following examination in an ED room. These included a complete blood count, complete metabolic profile, POC urine pregnancy screen, lipase, urinalysis, and COVID-19/influenza swab. Chest X-ray was obtained secondary to the shortness of breath, and Computed Tomography (CT) abdomen/pelvis with intravenous (IV) contrast was obtained secondary to the upper abdominal and RUQ tenderness to palpation. Acetaminophen 1000mg by mouth was given for her pain, ondansetron 4mg intravenous (IV) was given for her nausea, and 1 liter of 0.9% sodium chloride was given for presumed dehydration due to dry oral mucosa. Differential diagnoses included viral syndrome, gastritis, gastroenteritis, pancreatitis, cholecystitis, biliary colic, and pneumonia. The patient was at low risk for deep venous thrombosis (DVT) or pulmonary embolism (PE) with a Well’s score of 0. 

**Figure 1:**
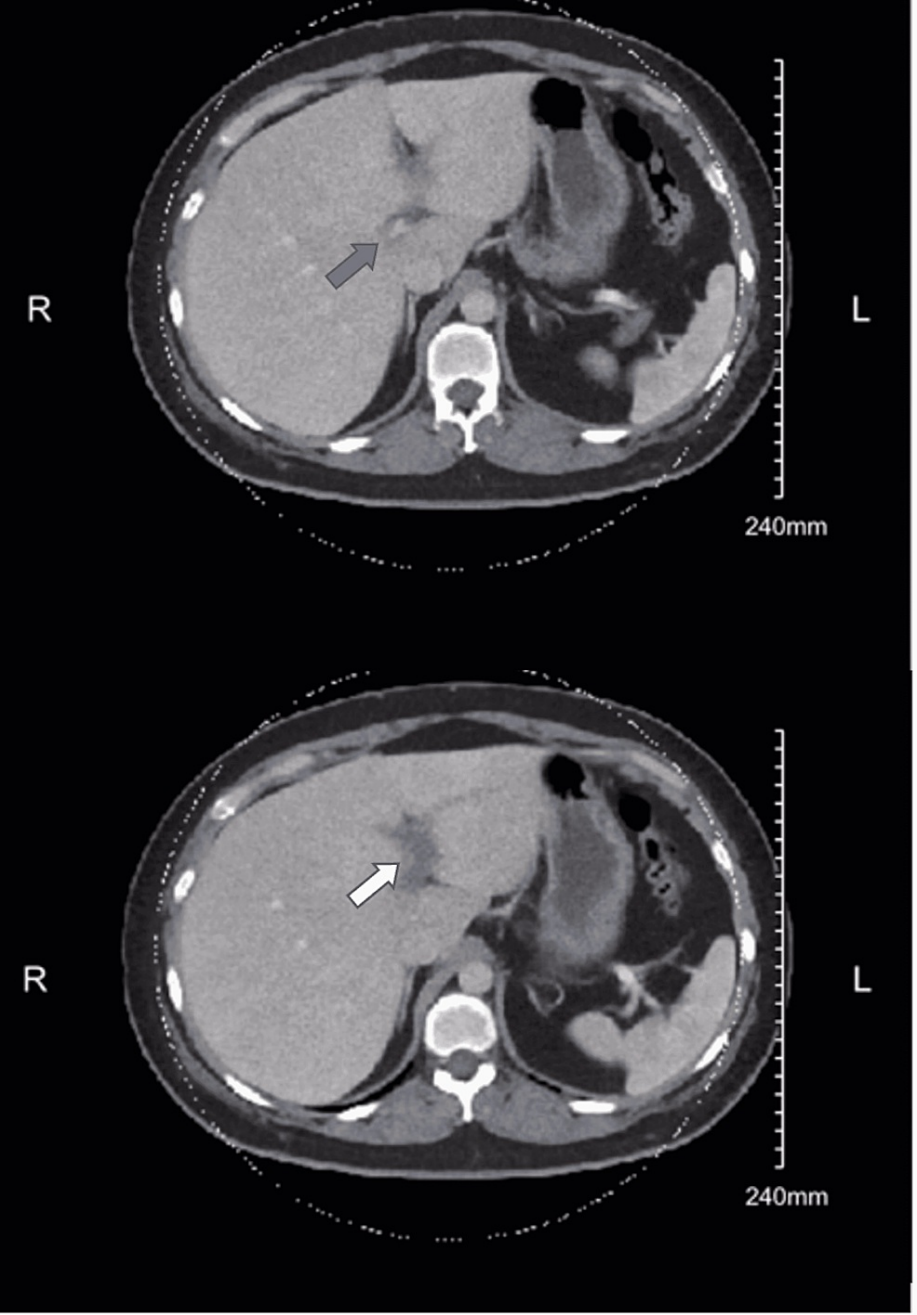
CT abdomen (axial view) Axial view of proximal (grey arrow) and distal portal vein thrombosis (white arrow)

**Figure 2:**
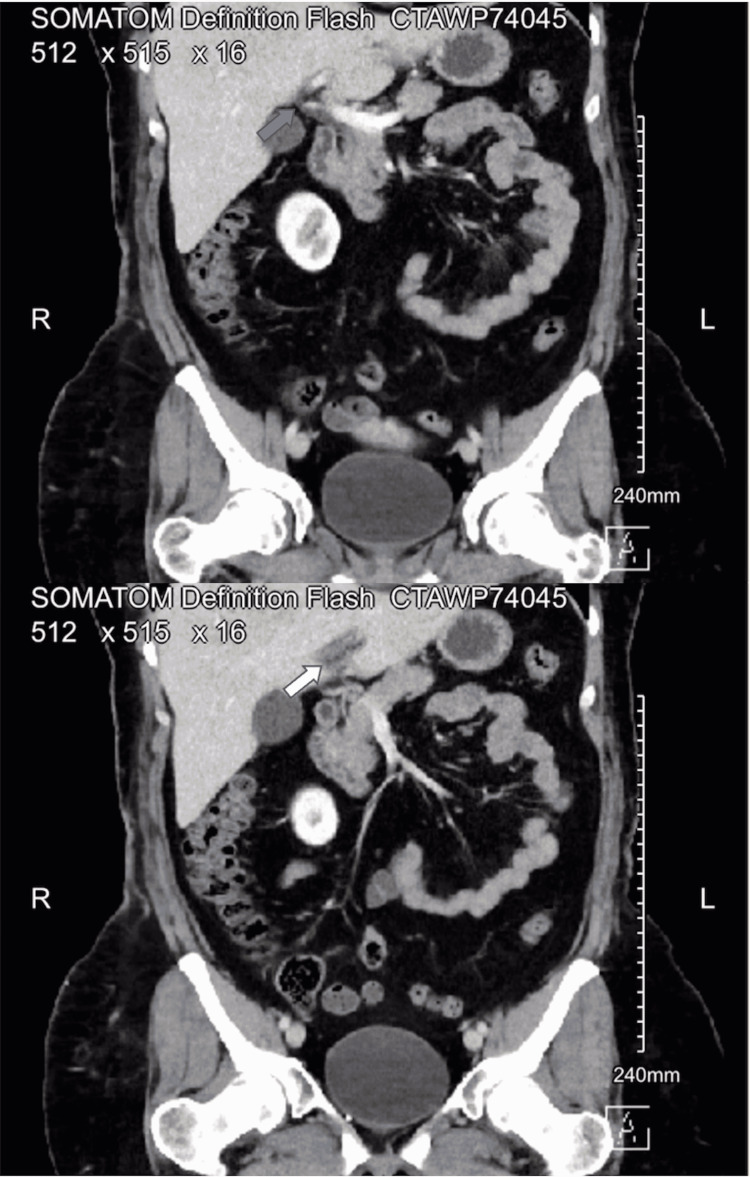
CT abdomen/pelvis (coronal view) Coronal view of proximal (grey arrow) and distal portal vein thrombosis (white arrow)

Lab work returned without significant abnormalities. Her thyroid-stimulating hormone had been taken 1.5 months prior and was WNL. Chest X-ray returned without significant abnormality. In the meantime, the patient continued to have pain in the upper abdomen, so a gastrointestinal (GI) cocktail consisting of magnesium hydroxide/aluminum hydroxide/simethicone was given. Shortly thereafter, CT abdomen/pelvis returned with the impression of left portal vein thrombosis (Figures 1, 2), recommended correlating for thrombophlebitis, and that there was no discrete hepatic abscess. 

**Figure 3:**
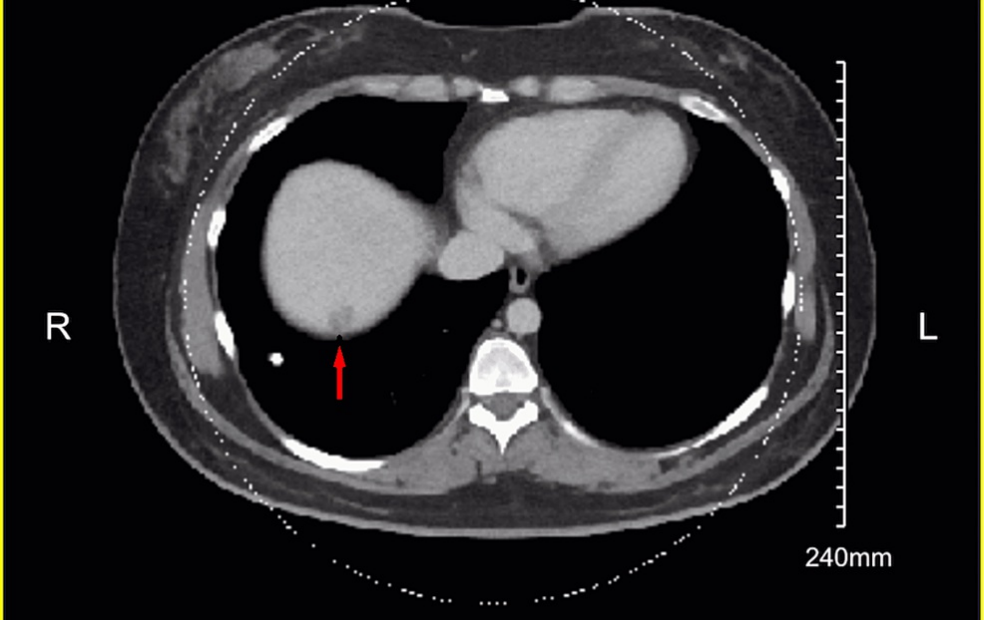
CT right hepatic dome of the liver Hypoattenuating focus in the right hepatic dome of the liver (red arrow)

CT also found a hypoattenuating focus in the right hepatic dome which was favored to be a hemangioma, although incompletely characterized (Figure 3). 

Hematology and liver service were consulted after the physician was notified by radiology about the CT results. At this time, the portal venous thrombosis was thought to be secondary to the COVID-19 vaccine vs. OCP due to the patient’s young age and no other risk factors for clotting. Liver service noted that the patient also had generalized pruritus intermittently since the onset of her symptoms. They recommended ordering Hepatitis A (HAV) IGG antibody, Hepatitis B surface antibody/antigen, Hepatitis C antibody, and Magnetic Resonance Imaging with the contrast of liver (to confirm hemangioma and exclude other etiologies). Hematology notes that the patient had mid-epigastric pain for four days and that deep breathing caused the pain to worsen. They stated that her mother was found to have a DVT at age 57, approximately one month after obtaining a COVID-19 vaccine, and had a thrombosis during cancer treatment in the past. Hematology recommended reporting the portal venous thrombosis on Vaccine Adverse Event Reporting System, sending *JAK V617F*, paroxysmal nocturnal hemoglobinuria flow, and antiphospholipid syndrome Ab testing (all returned without abnormality). Both labs from hematology and liver service’s recommendation were ordered prior to the patient being discharged. The patient was instructed to start Xarelto® (Janssen, Titusville, NJ) 15mg twice a day for 21 days, then start taking 20mg daily for 6-12 months. The patient was also instructed to avoid estrogen-containing OCPs going forward and to use progesterone-only OCPs or intrauterine devices. 

Hematology evaluated the patient approximately one month later. Labs recommended by liver service and hematology were significant for a reactive hepatitis A IgG antibody consistent with the patient's HAV immunization status. There were no other significant abnormalities. No change was made to the plan. 

## Discussion

Acute abdominal pain represents approximately 10% of all emergency department visits and has a wide differential diagnosis [4]. Although PVTs are a rare cause of abdominal pain, especially in young and healthy individuals, early diagnosis and intervention are crucial to prevent thrombus propagation and organ damage. A systematic review compiled case reports of 16 acute PVTs with an average symptom onset of 8.3 days post-COVID-19 vaccination. Of those cases, 75% were females with no liver disease. Only three patients had non-age-related risk factors for clotting, namely obesity (age 62), treated breast cancer (age 57), and Budd-Chiari syndrome and myeloproliferative disorder (age 46), indicating COVID-19 vaccines as a potential new etiology for portal thrombosis [5]. Of these cases, 87.5% received the ChAdOx1 nCoV-19 vaccine (Oxford-AstraZeneca) and 12.5% received Janssen (Johnson & Johnson) [5], with no literature found attributing PVTs to Moderna and very few case reports involving young patients, making this a notable presentation.

Due to the unclear etiology of this PVT, numerous literature searches concerning the relationship between pathologic clotting and each potential risk factor were conducted. The most notable prothrombotic predisposition of this patient was her use of combined OCPs, which only increases the risk for venous thromboembolism (VTE) from 0.01-0.05% to 0.03-0.09% [6], making the likelihood of causing a PVT extremely low [6]. The patient's obesity is considered a risk factor for VTE, yet in a study with nearly 70,000 obese cirrhotics, there was no increased risk of PVT compared to their non-obese counterparts [7]. Similarly, this patient's reactive HAV IgG indicates immunization or past HAV infection, with no PVTs related to HAV found in the literature [8]. Per the patient, the history of VTE in her mother was during active malignancy and not due to any inheritable risk factor.

There is one case report that details a 35-year-old female who used OCPs and developed a PVT six days after the ChAdOx1 nCoV-19 vaccine. She presented in a unique way with complaints of headache, nausea, and vomiting but no abdominal tenderness [9]. Oxford-AstraZeneca and Johnson & Johnson vaccines, both adenovirus-based vaccines, have been linked to a novel syndrome known as vaccine-induced immune thrombotic thrombocytopenia (VITT). The estimated incidence of VITT is 1:50,000 in patients younger than 50 [10]. Although the mechanism is unclear, there is speculation that the adenovirus triggers pathologic clotting in certain individuals.

Our patient's PVT, which occurred five days post-COVID vaccination, correlates with the timeline of all other suspected vaccine-induced PVTs in the literature. Considering our patient's unremarkable workup for coagulopathies and lack of convincing evidence supporting a known etiology, it is reasonable to consider this case as an example of vaccine-induced portal vein thrombosis.

## Conclusions

Although portal vein thrombosis is a rare pathology, physicians should know the signs, symptoms, and risk factors. The more prevalent risk factors include cirrhosis, prothrombotic disorders, and neoplasms. Once a PVT has been diagnosed without a known etiology, physicians should order a hematology work-up including prothrombin time/international normalized ratio (PT/INR), *JAK V617F*, paroxysmal nocturnal hemoglobinuria flow, and antiphospholipid syndrome Ab testing, start the patient on anticoagulants, and take a thorough vaccine history.

In this case report, we have tried to supply evidence of a novel COVID vaccination causing acute PVT. With the average onset of symptoms being eight days in case reports in the literature, our patient's clinical presentation five days post-vaccination is highly suspicious for a vaccine-induced thrombosis. Several formulations, namely Oxford-AstraZeneca and Johnson & Johnson, have been linked to PVT formation, and we provide a case that gives evidence to the first reported mRNA-based PVT. There is a growing literature supporting vaccine-induced thromboses; therefore, once common causes of PVT have been ruled out, consider COVID-19 vaccines as a potential etiology in the acute setting. While the COVID-19 vaccine provides more benefit than harm, unusual medical complications that novel vaccines may cause should be reported to the Vaccine Adverse Event Reporting System to increase monitoring and awareness of vaccine-related adverse effects.

Further exploration is needed to determine the mechanisms and individual risk factors that cause thrombosis after vaccine administration. We encourage physicians to be aware of the possibility of this etiology and to take thorough COVID-19 vaccination histories in those diagnosed with clotting pathologies to better understand the relationship between pathologic clotting and COVID-19 vaccines.
